# Prospective Head-to-Head Comparison of ^18^F-PSMA PET/CT and ^18^F-NaF PET/CT for Assessing Bone Metastases in 160 Patients with Newly Diagnosed High-Risk Prostate Cancer

**DOI:** 10.2967/jnumed.124.268275

**Published:** 2025-02

**Authors:** Claus Madsen, Dan Fuglø, Maria Pedersen, Rikke Broholm, Peter B. Østergren, Rasmus Bisbjerg, Per Kongsted, Kayalvili Nielsen, Christian Haarmark, Helle Zacho

**Affiliations:** 1 Department of Nuclear Medicine, Copenhagen University Hospital Herlev and Gentofte, Herlev, Denmark;; 2 Department of Urology, Copenhagen University Hospital Herlev and Gentofte, Herlev, Denmark;; 3 Department of Clinical Medicine, Copenhagen University, Copenhagen, Denmark;; 4 Department of Oncology, Copenhagen University Hospital Herlev and Gentofte, Herlev, Denmark;; 5 Department of Radiology, Copenhagen University Hospital Herlev and Gentofte, Herlev, Denmark; and; 6 Department of Nuclear Medicine, Aalborg University Hospital, Aalborg, Denmark

**Keywords:** prostate cancer, bone metastases, PSMA PET/CT, NaF PET/CT, diagnostic accuracy

## Abstract

Prostate-specific membrane antigen (PSMA) PET/CT is increasingly used for primary staging in prostate cancer (PC), mainly because of its improved accuracy in detecting lymph node metastases compared with conventional imaging. However, the diagnostic benefit of PSMA PET/CT for detecting bone metastases is less well established. This study compares the diagnostic accuracy of ^18^F-PSMA PET/CT and ^18^F-NaF PET/CT for detecting bone metastases in patients newly diagnosed with PC. **Methods:** This prospective study included patients with histologically confirmed high-risk PC. All participants were referred from the department of urology to ^18^F-NaF PET/CT and underwent ^18^F-PSMA PET/CT within 3 weeks. Images were reviewed by 2 nuclear medicine physicians unaware of the results of the other imaging modality. Presence or absence of bone metastases and number of metastatic lesions were recorded. A reference standard was established at the patient level based on agreement between the 2 imaging modalities. In cases of concordance, both modalities were deemed correct. In cases of discordance, additional follow-up scans were performed. Diagnostic performance metrics, including sensitivity, specificity, and accuracy, were calculated. **Results:** In total, 160 participants were included. Sensitivity, specificity, and accuracy for detecting bone metastases at the patient level were 0.98, 0.99, and 0.99, respectively, for ^18^F-PSMA PET/CT, and 0.91, 1.00, and 0.97, respectively, for ^18^F-NaF PET/CT. No significant differences were found. The concordance rate of bone metastases between ^18^F-NaF and ^18^F-PSMA PET/CT at the patient level was observed in 154 patients (96.3%). ^18^F-PSMA PET/CT tended to identify more bone metastases per patient than ^18^F-NaF PET/CT. **Conclusion:** Both ^18^F-NaF and ^18^F-PSMA PET/CT exhibit high diagnostic accuracy for detecting bone metastases in newly diagnosed high-risk PC patients. ^18^F-PSMA PET/CT may detect additional metastatic lesions compared with ^18^F-NaF PET/CT. Subsequent ^18^F-NaF PET/CT may be redundant if no bone metastases are found on ^18^F-PSMA PET/CT.

## Visual Abstract

**Figure:**
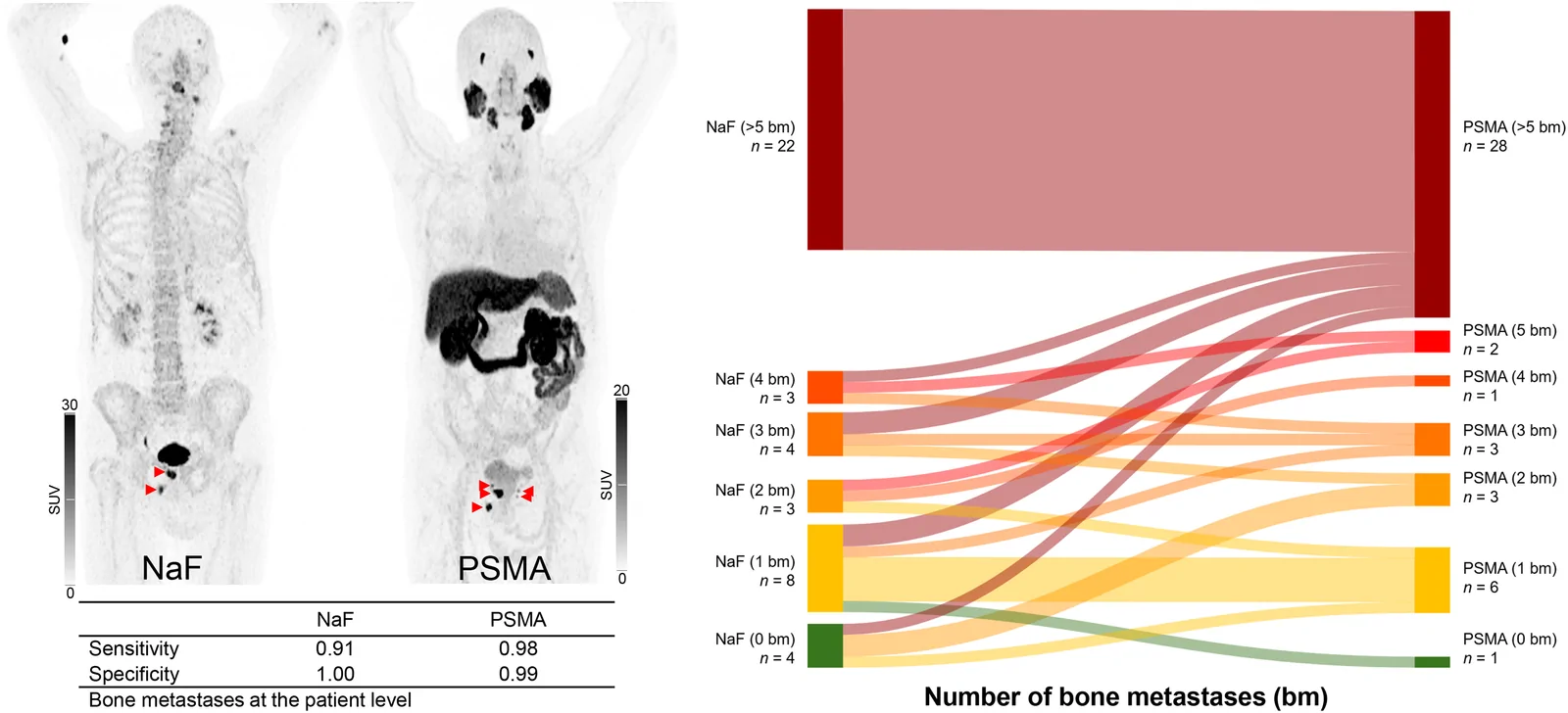

Prostate cancer (PC) is among the most frequently diagnosed malignancies globally (1). It predominantly spreads to lymph nodes and bones. Accurate initial staging is essential for selecting appropriate treatment. Imaging modalities typically include bone scintigraphy and CT (2,3). However, CT has limited sensitivity for detecting lymph node metastases (4).

Prostate-specific membrane antigen (PSMA) ligands are used in PET and recognized for their potential in imaging metastatic PC. The ligand binds to a membrane glycoprotein that is upregulated on the surface of PC cells (5), serving as a direct marker of disease distribution, including bone involvement.

In contrast, imaging with bone-seeking agents, such as diphosphonates used in bone scintigraphy and ^18^F-NaF used in ^18^F-NaF PET/CT, reflects osteoblastic bone reformation. This reformation is influenced by, among other factors, metastases from PC. ^18^F-NaF PET/CT offers several advantages over bone scintigraphy, including higher spatial resolution and rapidly acquired tomographic imaging of the entire area of interest.

Several studies have demonstrated improved diagnostic accuracy of PSMA PET/CT for detecting lymph node metastases compared with CT and MRI. (6*–*8). Consequently, the European Association of Urology now recommends PSMA PET/CT for initial staging in patients with high-risk PC (3). Although multiple studies suggest that PSMA PET/CT is superior to bone scintigraphy for detecting bone metastases (9), the efficacy of the PSMA radioligand compared with ^18^F-NaF for skeletal evaluation in initial staging remains uncertain. Indeed, no large prospective studies have compared PSMA PET/CT with ^18^F-NaF PET/CT in patients with newly diagnosed disease.

The aims of this study were to compare ^18^F-PSMA PET/CT and ^18^F-NaF PET/CT for the assessment of bone metastases in patients with newly diagnosed high-risk PC and to determine whether ^18^F-NaF PET/CT can be omitted when no bone metastases are found on ^18^F-PSMA PET/CT.

## MATERIALS AND METHODS

### Participants

This prospective, single-center study included consecutive patients with histologically proven PC, categorized as high-risk according to the D’Amico criteria (T stage ≥ cT2c, International Society of Urological Pathology grade group ≥ 4, or prostate-specific antigen > 20 ng/mL) (10). T stage was based on digital rectal examination. Patients with a high clinical suspicion of PC (high prostate-specific antigen or T2c-T4 tumor) were often referred for metastatic evaluation before definitive histology of the prostate biopsies and could thus be included in the study. Patients were excluded from the analysis if the final histopathologic results did not confirm PC.

All patients underwent ^18^F-NaF PET/CT as part of the standard diagnostic work-up at Copenhagen University Hospital Herlev and Gentofte. Participant enrollment took place from May 4, 2021, to February 20, 2024.

Patients were excluded if they were diagnosed with a nonprostate malignancy within the last 5 y or during the diagnostic work-up for PC (except for basal cell or squamous cell skin cancer), had commenced androgen deprivation therapy, had severe obesity, or could not cooperate.

Informed oral and written consent from the participants was obtained at the Department of Nuclear Medicine before the clinical ^18^F-NaF PET/CT scan. The ^18^F-PSMA PET/CT was performed as a study-specific procedure within 3 wk of the ^18^F-NaF PET/CT.

### ^18^F-NaF PET/CT

^18^F-NaF PET/CT was conducted as part of daily clinical practice. A mean dose of 199 MBq (SD, ±3 MBq; range, 172–207 MBq) was injected 30 min before image acquisition. PET/CT was performed using a Biograph mCT (Siemens Healthineers). Images were acquired from the top of the skull to above the knees, with an acquisition time of 1 min per bed position. The ^18^F-NaF PET was combined with either low-dose CT (tube current–time product of 40 mAs with dose modulation, 120 kV, pitch of 0.8, and 5-mm slice thickness) or diagnostic CT (tube current–time product of 180 mAs with dose modulation, 120 kV, pitch of 0.8, and 3-mm slice thickness) if soft-tissue evaluation was indicated. In diagnostic CT, iodinated contrast medium was infused if tolerated. The choice between low-dose and diagnostic CT was made at the discretion of the referring urologist.

### ^18^F-PSMA PET/CT

The ^18^F-labeled ^18^F-PSMA-1007 ligand was used for the study. A mean dose of 256 MBq (SD, ±14 MBq; range, 210–316 MBq) was injected 60 min before image acquisition on a Biograph mCT PET/CT scanner. Images were acquired from the top of the skull to above the knees, with an acquisition time of 3 min per bed position. The PET scan was combined with a diagnostic CT scan.

### Image Analysis

The image analyses of ^18^F-NaF and ^18^F-PSMA PET/CT were performed independently by 2 board-certified nuclear medicine physicians, each with extensive experience in interpreting ^18^F-NaF PET/CT and ^18^F-PSMA PET/CT. The readers did not know the results of the other imaging modality or the clinical information, except for the diagnosis of high-risk PC.

At the patient level, whether the patient had bone metastases was recorded. The level of confidence was noted as either no doubt or with doubt; an inconclusive scan was not an available option. At the lesion level, the number of bone metastases was recorded as 1, 2, 3, 4, 5, or more than 5. Factors influencing the interpretation toward bone metastasis included ^18^F-PSMA uptake equal to or above the spleen (11), well-defined lesion borders, involvement of cancellous bone, an osteoblastic appearance on CT, and multiple lesions with metastatic characteristics.

The treating physicians did not know the results of the ^18^F-PSMA PET/CT, as it was not part of the standard diagnostic work-up at our institution. However, if requested by the treating physicians, a report of the ^18^F-PSMA PET/CT scan was provided.

### Reference Standard

**FIGURE 1.**
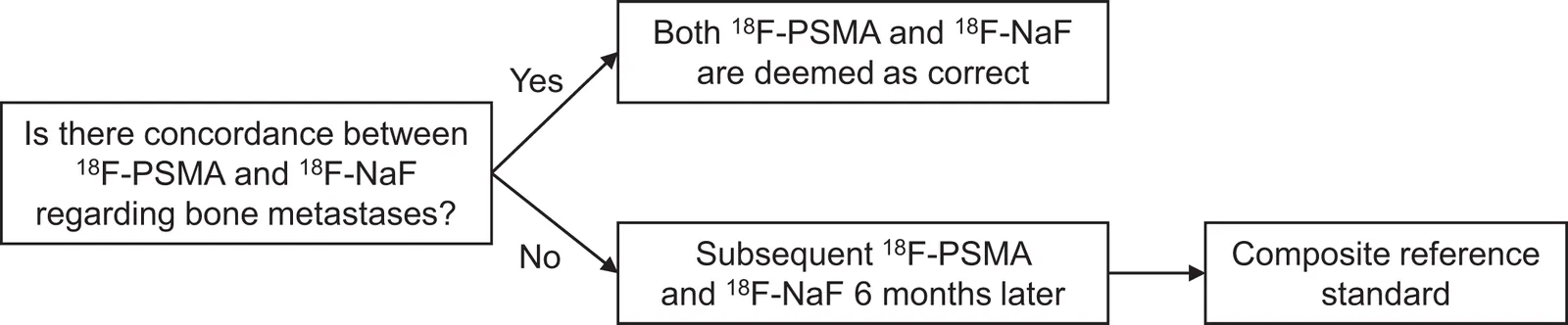
Flowchart illustrating process of achieving final diagnosis of bone metastases on patient level.

Histopathologic reference standards for the presence or absence of bone metastases were not available for ethical and practical reasons in most cases. If neither ^18^F-NaF PET/CT nor ^18^F-PSMA PET/CT revealed any bone metastases, the patient was categorized as having no bone metastases. If both modalities agreed on the location of at least 1 bone metastasis, the patient was categorized as having bone metastases. In cases of discordance between ^18^F-NaF and ^18^F-PSMA PET/CT, both scans were repeated once at least 6 mo after the initial ^18^F-PSMA PET/CT scan (Fig. 1). The subsequent ^18^F-NaF and ^18^F-PSMA PET/CT, along with all available clinical data including treatment and biochemical data, were included in a composite reference standard, determined by a multidisciplinary panel consisting of a urologist, an oncologist, and 2 nuclear medicine specialists.

### Statistics

Statistical analyses were performed using RStudio, version 4.4.0 (Posit Software). Patients’ clinical characteristics are reported as descriptive statistics, including mean or median, SD, and range. For each imaging modality, the sensitivity, specificity, positive predictive value, negative predictive value, and accuracy, along with 95% confidence intervals, were calculated on a patient level using the epiR package. The McNemar test was performed to compare diagnostic accuracies using the DTComPair package. The threshold for rejecting no difference between the 2 imaging methods was 0.05.

All data were collected in the REDCap database (Vanderbilt University). The Sankey diagram was made with SankeyMATIC.

### Ethics

This study complied with the Helsinki II Declaration. All patients provided written informed consent to participate. The study protocol was approved by the regional ethics committee (approval H-20060829) and the Danish Data Protection Agency.

## RESULTS

### Study Population

**FIGURE 2.**
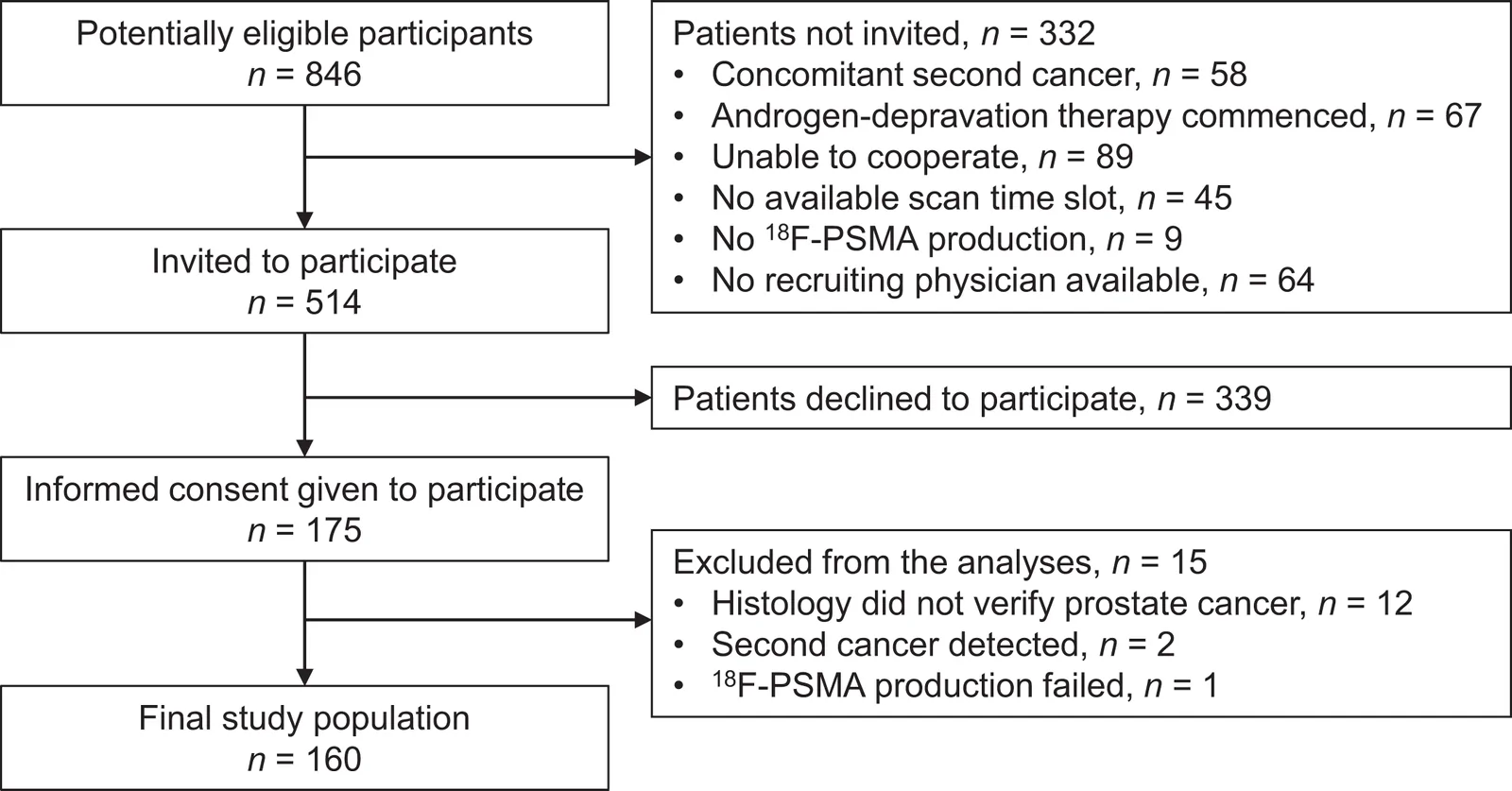
Flowchart illustrating inclusion process of study participants.

In total, 846 potentially eligible participants were screened, and 332 were not invited to participate because of other types of active cancer, initiation of androgen-deprivation therapy before the scan, or other reasons (Fig. 2). Thus, 514 patients were invited to participate, and 175 accepted the invitation. Fifteen patients were excluded from the data analysis: 12 had a benign final histopathologic result from the prostate biopsies, 2 were diagnosed with an additional malignancy during the diagnostic work-up for PC, and one did not undergo ^18^F-PSMA PET/CT because of ^18^F-PSMA production failure. The final study population comprised 160 patients with newly diagnosed high-risk PC (Table 1). All were diagnosed with acinar adenocarcinoma of the prostate. One patient had a component of small cell neuroendocrine carcinoma, which made up 25% of the tumor. On average, 10 d (range, 1–21 d) elapsed between the routine ^18^F-NaF PET/CT and the ^18^F-PSMA PET/CT.

**TABLE 1.** Demographic Data of Study Population

Demographic	Data
Mean age (y)	72 (range, 54–88)
Median PSA (ng/mL)	35 (range, 2.3–7,701)
PSA category (*n*)	
<10 ng/mL	28 (18%)
10–20 ng/mL	22 (14%)
20.1–49.9 ng/mL	50 (31%)
50–99.9 ng/mL	28 (18%)
≥100 ng/mL	32 (20%)
cT-stage (*n*)	
Tx	9 (6%)
T1	12 (8%)
T2a-T2b	13 (8%)
T2c	21 (13%)
T3	83 (52%)
T4	22 (14%)
ISUP grade group (*n*)	
1–2	23 (14%)
3	57 (36%)
4–5	80 (50%)

PSA = prostate-specific antigen; cT-stage = clinical tumor stage; ISUP = International Society of Urological Pathology.

### Patient-Based Diagnostic Accuracy Measurements

**FIGURE 3.**
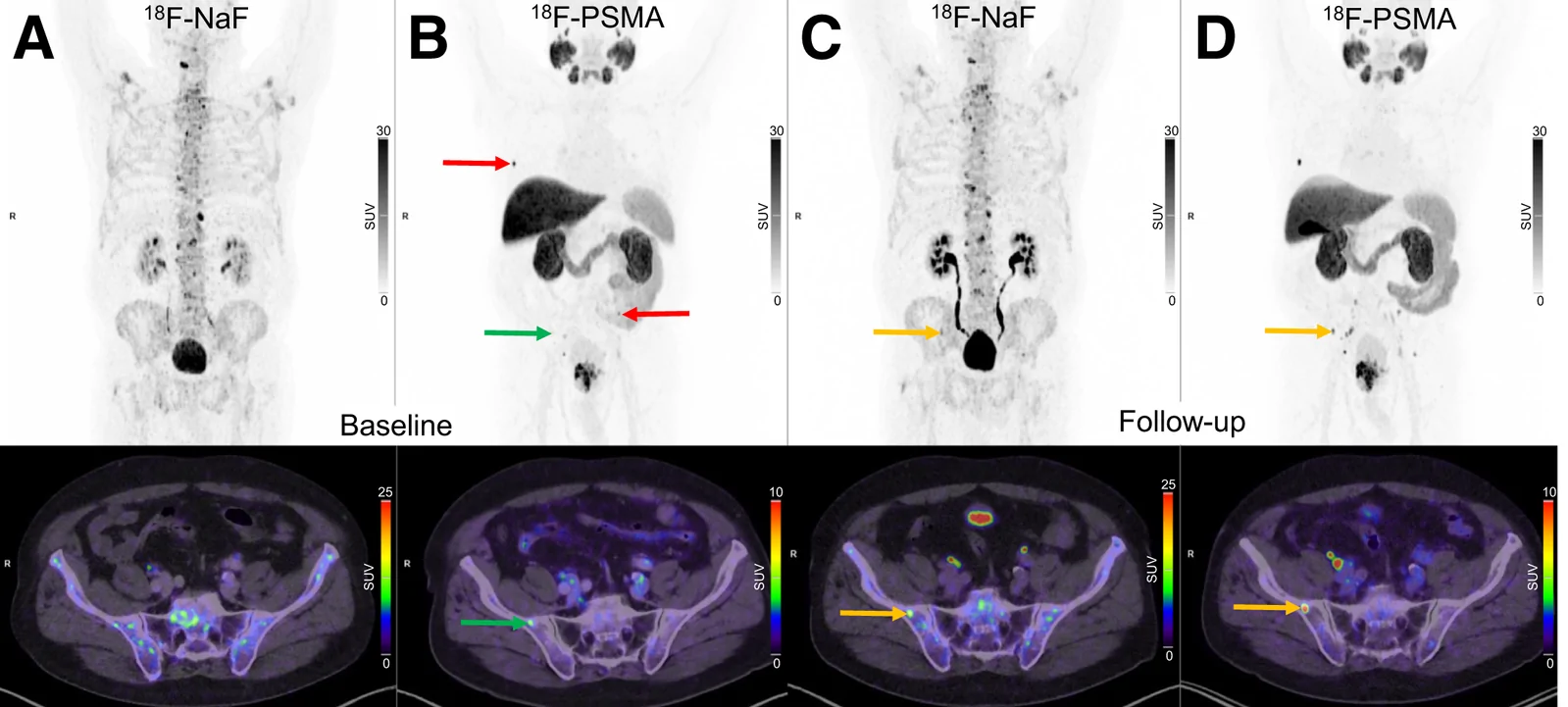
Patient with discordant findings on baseline ^18^F-NaF and ^18^F-PSMA PET/CT. (A) Maximum-intensity projection and axial projection of ^18^F-NaF PET/CT scan with no evidence of bone metastases. (B) ^18^F-PSMA PET/CT scan showing 2 lesions, one in left ilium and another in right costa 6, which were suspected of being bone metastases (red arrows). Third faint lesion in right ilium was not interpreted as bone metastasis (green arrows). Because of absence of metastatic lesions on ^18^F-NaF PET/CT (conventional scan), patient was observed, without treatment. (C and D) Follow-up scans conducted 6 months later revealed detectable uptake in lesion in right ilium on ^18^F-NaF PET/CT and increasing uptake on ^18^F-PSMA PET/CT (orange arrows). There are multiple ^18^F-PSMA-avid lymph nodes on follow-up scan.

According to the reference standard, 44 patients (27.5%) had bone metastases. In 154 of 160 patients (96.3%), there was concordance between ^18^F-PSMA and ^18^F-NaF PET/CT regarding the presence or absence of bone metastases at the patient level. Both scans were thus deemed correct in these cases. ^18^F-PSMA PET/CT differed from ^18^F-NaF PET/CT in 6 patients (3.8%), who subsequently underwent additional ^18^F-PSMA and ^18^F-NaF PET/CT 6 mo later. Of all available imaging and patient records, ^18^F-NaF PET/CT correctly classified 2 patients, whereas ^18^F-PSMA PET/CT correctly classified the remaining 4 patients (Table 2; Fig. 3). Sensitivity, specificity, and accuracy for detecting bone metastases at the patient level were 0.98, 0.99, and 0.99, respectively, for ^18^F-PSMA PET/CT and 0.91, 1.00, and 0.97, respectively, for ^18^F-NaF PET/CT (Table 3). There was no significant difference in diagnostic performance between ^18^F-PSMA and ^18^F-NaF PET/CT (*P* = 0.18, 0.31, and 0.68, respectively) according to the McNemar test.

**TABLE 2.** Patients with Discordant Findings on ^18^F-NaF PET/CT and ^18^F-PSMA PET/CT and with Repeated Scans 6 Months Later

Patient	PSA	cT-stage	ISUP	Treatment between baseline and follow-up scans	Bone metastases on ^18^F-NaF PET/CT	Bone metastases on ^18^F-PSMA PET/CT	Bone metastases, reference standard*
36	64	T1c	3	EBRT to prostate and pelvic lymph nodes and ADT with LHRH agonist with curative intent	Yes—one lesion near right ischial tuberosity	No—only low uptake near right ischial tuberosity	Yes—increasing uptake on follow-up ^18^F-NaF and ^18^F-PSMA; increasing sclerotic appearance
50	89	T4	4	ADT with LHRH agonist. Bicalutamide for 30 d	No	Yes—high uptake in left ilium and low uptake in sacrum	Yes—no change on follow-up ^18^F-NaF and ^18^F-PSMA; from all available data, disseminated bone disease on baseline is assumed
79	104	T3b	5	ADT with LHRH agonist	No	Yes—multiple small bone metastases	Yes—some lesions (left costa 1 and Th12) become osteoblastic on follow-up CT
86	20	T3a	5	Small cell component; treated with ADT (LHRH agonist) and carboplatin and etoposide	No	Yes—small lesion in left femoral neck	No—^18^F-PSMA uptake becomes slightly faint on follow-up scan; lack of visible ^18^F-NaF uptake on follow-up is unchanged; no osteoblastic formation
117	4.8	T2c	4	EBRT to prostate and pelvic lymph nodes and ADT with LHRH agonist with curative intent	No—uptake in left ilium interpreted benign	Yes—high uptake in left ilium	Yes—lesion becomes faint on follow-up ^18^F-NaF and ^18^F-PSMA; small osteoblastic formation
190	25	T2a	4	Watchful waiting	No	Yes—high uptake in left ilium and right costa 6	Yes—new lesion in right ilium on follow-up ^18^F-NaF and ^18^F-PSMA; multiple new ^18^F-PSMA-avid lymph nodes on follow-up

* Reference standard is determined in multidisciplinary team conference and is based on baseline and follow-up PET/CT scans and available clinical data.

PSA = prostate-specific antigen; cT-stage = clinical tumor stage; ISUP = International Society of Urological Pathology; EBRT = external-beam radiation therapy; ADT = androgen deprivation therapy; LHRH = luteinizing hormone–releasing hormone.

**TABLE 3.** Diagnostic Performances at Patient Level

Imaging modality	^18^F-PSMA PET/CT	^18^F-NaF PET/CT
Sensitivity	0.98 (0.88–1.00)	0.91 (0.78–0.97)
Specificity	0.99 (0.95–1.00)	1.00 (0.97–1.00)
PPV	0.98 (0.88–1.00)	1.00 (0.91–1.00)
NPV	0.99 (0.95–1.00)	0.97 (0.92–0.99)
Accuracy	0.99 (0.96–1.00)	0.97 (0.94–0.99)
True-positive	43 (27%)	40 (25%)
False-positive	1 (1%)	0 (0%)
True-negative	115 (72%)	116 (73%)
False-negative	1 (1%)	4 (3%)

PPV and NPV = positive and negative predictive value, respectively.

Data in parentheses are 95% CI or percentage.

### Number of Bone Metastases

**FIGURE 4.**
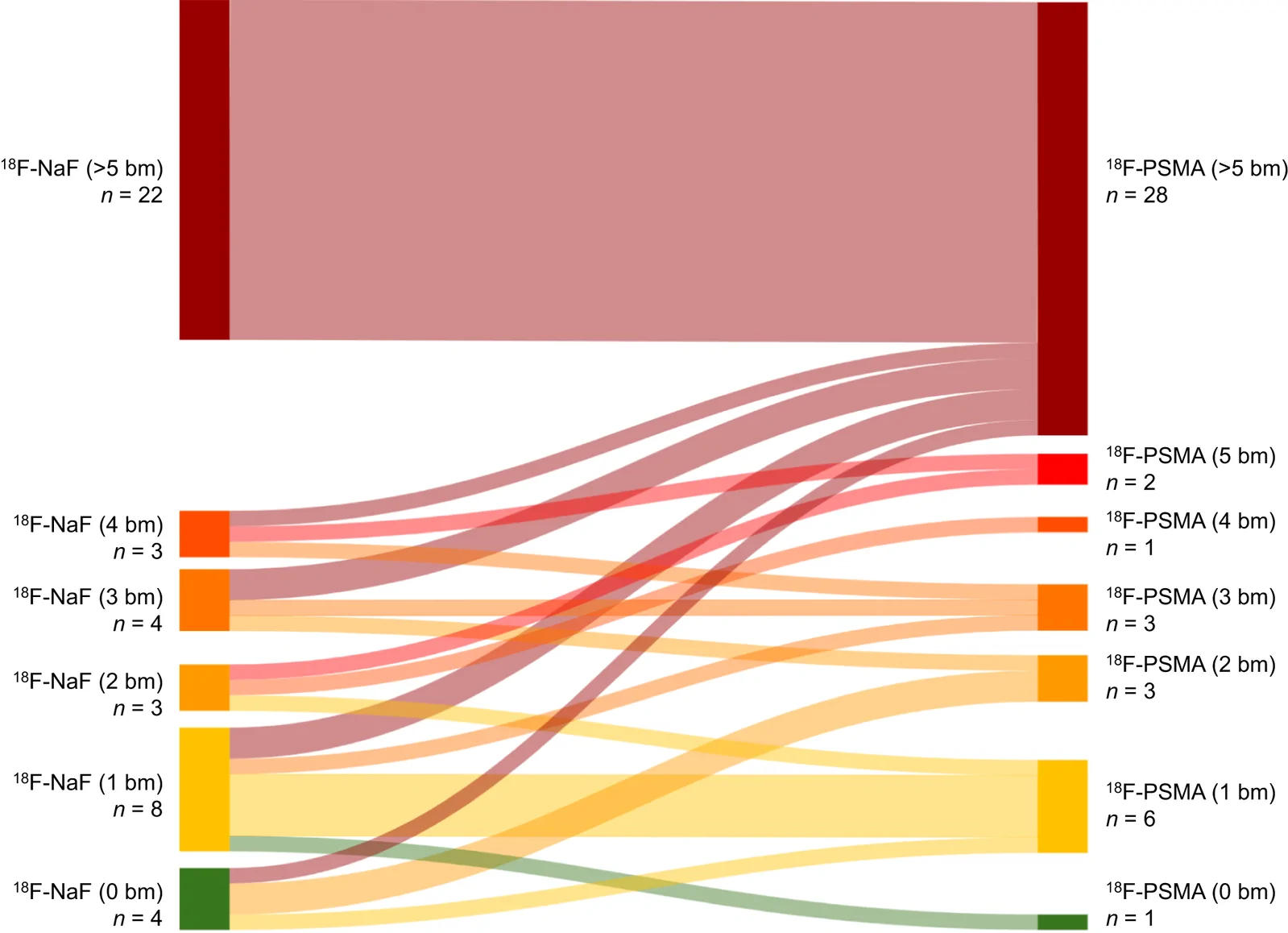
Sankey diagram of number of bone metastases detected by ^18^F-NaF PET/CT and ^18^F-PSMA PET/CT in 44 patients with bone metastases according to reference standard. bm = bone metastases.

In each scan, the number of metastases was assessed (1–5 or >5). Generally, ^18^F-PSMA PET/CT revealed more bone metastases than ^18^F-NaF PET/CT (Fig. 4). In 8 patients, ^18^F-NaF PET/CT identified a single metastatic lesion. Of these, anatomically correlated lesions were also detected on 7 ^18^F-PSMA PET/CT scans; however, in 1 patient, ^18^F-PSMA PET/CT incorrectly interpreted the lesion (located in the ischial tuberosity) as benign. Conversely, ^18^F-PSMA PET/CT detected additional bone metastases in 3 patients compared with ^18^F-NaF PET/CT, revealing 2 additional bone metastases in 1 patient and polymetastatic disease (>5) in 2 patients.

^18^F-PSMA PET/CT identified more patients with polymetastatic disease (*n = 28*) than did ^18^F-NaF PET/CT (*n = 22*). Among patients with oligometastatic (1–5) bone involvement according to ^18^F-NaF PET/CT (*n = 18*), the pelvis was involved in most cases (*n = 14*, 78%). The 4 patients (22%) with exclusively extrapelvic bone involvement each had only 1 metastatic lesion detected.

### Level of Confidence

The reader of ^18^F-NaF PET/CT was confident about the presence or absence of bone metastases at the patient level in 139 (87%) of 160 scans (Table 4). The reader of ^18^F-PSMA PET/CT was confident in 143 (89%) scans. The level of confidence was highest for both modalities (100%) when 3 or more bone metastases were detected. Conversely, when only 1 suggestive lesion was detected, the reader of ^18^F-NaF PET/CT was confident in 1 of 8 scans (13%) whereas the reader of ^18^F-PSMA PET/CT was confident in 4 of 7 scans (57%).

**TABLE 4.** Proportion of Scans with Confident Conclusions, Stratified by Number of Bone Metastases

Bone metastases (*n*)	Scans with confident conclusions	
	NaF	PSMA
0	108/120 (90%)	103/116 (89%)
1	1/8 (13%)	4/7 (57%)
2	1/3 (33%)	2/3 (67%)
3–5	7/7 (100%)	6/6 (100%)
>5	22/22 (100%)	28/28 (100%)
Total	139/160 (87%)	143/160 (89%)

## DISCUSSION

This study was, to the best of our knowledge, the first large prospective study comparing ^18^F-PSMA and ^18^F-NaF PET/CT for detecting bone metastases in a homogeneous cohort of patients at the primary staging of PC. The study revealed high concordance between the 2 modalities for detecting bone metastases, with crude agreement in 96% of the patients. There was a trend for ^18^F-PSMA PET/CT to detect more patients with bone metastases and to identify a higher number of lesions in metastatic patients. However, both imaging modalities demonstrated high diagnostic accuracy for detecting bone metastases.

Few studies have compared the diagnostic accuracy of PSMA radioligands and ^18^F-NaF PET/CT for detecting bone metastases. Dyrberg et al. examined 55 patients across various stages of PC. Both ^18^F-NaF and ^68^Ga-PSMA PET/CT demonstrated high diagnostic accuracy, ranging from 96% to 100% (12). Zacho et al. found comparably high sensitivity and specificity for both modalities in 68 patients with biochemically recurrent PC (13).Two smaller studies, including patients with metastatic PC (14) and those after curative treatment (15), showed promising results for both modalities.

Bone scintigraphy has been more extensively compared with PSMA PET/CT. A prospective study of 113 patients with newly diagnosed PC showed that ^68^Ga-PSMA PET/CT had significantly higher sensitivity and accuracy than bone scintigraphy (16). Other studies have confirmed the improved sensitivity and specificity of ^68^Ga-PSMA PET/CT in PC patients at different stages (17*–*20). The large prospective proPSMA study also found that ^68^Ga-PSMA PET/CT had significantly higher accuracy for detecting distant metastases, although bone metastases were not assessed separately (21). In conclusion, the sensitivity of PSMA PET/CT is superior to that of bone scintigraphy in patients with PC, both at primary staging and at later disease stages.

We found a high concordance between ^18^F-PSMA and ^18^F-NaF PET/CT at the patient level, but ^18^F-PSMA PET/CT detected bone metastases in 4 additional patients. Moreover, ^18^F-PSMA PET/CT tended to identify more bone metastases in the individual patients. This finding opposes a study by Fourquet et al., who compared ^18^F-DCFPyL and ^18^F-NaF PET/CT in 61 men with known metastatic disease (22). ^18^F-NaF PET detected significantly more bone metastases than ^18^F-DCFPyL. However, the cohort comprised patients at different disease stages, and the difference between the 2 ligands was particularly clear for patients receiving treatment. The PSMA radioligands serve as direct markers of metastatic bone disease, whereas ^18^F-NaF reflects increased bone turnover, often a consequence of bone metastases from PC. This suggests that PSMA PET/CT might detect early metastatic presence in the bone marrow, followed by osteoblastic activity visible on ^18^F-NaF PET, and later by sclerotic bone formation on CT.

The low discordance rate of 3.8% in our study aligns with findings from a retrospective study involving a subset of patients who underwent ^68^Ga-PSMA and ^18^F-NaF PET/CT for primary staging (23). Three of 80 patients (3.8%) had bone metastases detected exclusively by ^68^Ga-PSMA PET/CT. Similar to our study, there was a trend indicating that ^68^Ga-PSMA PET/CT identified more metastatic bone lesions than did ^18^F-NaF PET/CT.

In our study, only 1 ^18^F-PSMA PET/CT scan was false-positive, showing a small lesion in the femoral neck. Unspecific bone uptake of ^18^F-PSMA-1007, used in this study, is well known (24), particularly in the ribs and pelvis (25). In patients with no detected bone metastases, the reviewers of ^18^F-PSMA and ^18^F-NaF PET/CT were uncertain in 11% and 10% of the scans, respectively, mostly because of unspecific bone uptake. The high proportion of unspecific bone uptake on ^18^F-PSMA PET/CT has been described as a major pitfall compared with ^68^Ga-PSMA-11 PET/CT (26). To minimize the risk of false positives, the present study involved the criterion that at least 1 bone metastasis should be anatomically correlated between ^18^F-PSMA and ^18^F-NaF PET/CT. We found a high specificity at the patient level. Thus, ^18^F-PSMA-1007 PET/CT is excellent for evaluating osseous involvement, but careful and experienced reading is crucial.

In both modalities, the lowest proportion of scans with a confident reading was observed when only 1 bone metastasis was detected. Treatment decisions depend on the diagnosis of bone metastases and whether oligo- or polymetastatic bone involvement is present (3). Indeed, diagnosing oligometastatic bone disease remains challenging. However, when 3 or more bone metastases were detected, the readers of both modalities felt confident in the diagnosis.

A limitation of the study was that only 19% of the potentially eligible participants were included. The ^18^F-PSMA PET/CT scans were anonymized and used solely for study purposes without remuneration. Consequently, selection bias may have occurred.

Another limitation was the single-center setup. Confirmation of the high diagnostic accuracies is warranted. Additionally, the cohort included patients with both curative and noncurative PC, and thus many of the patients had high prostate-specific antigen levels. The high concordance between ^18^F-PSMA and ^18^F-NaF PET/CT could be due to the high proportion of polymetastatic bone disease. Fewer bone metastases led to less reviewer confidence, increasing the risk of errors.

An additional limitation is the absence of a histopathologic reference standard. When no bone metastases were detected on either ^18^F-PSMA or ^18^F-NaF PET/CT, both modalities were deemed correct, which could mean that both might be false-negative because of micrometastatic bone involvement. Conversely, both imaging modalities could also have been false-positive, misinterpreting benign lesions as bone metastases. Indeed, tracer uptake may be increased in various benign conditions, such as fractures, Paget disease, and fibrous dysplasia in both ^18^F-PSMA PET (27) and ^18^F-NaF PET (28). Additionally, unspecific bone uptake of ^18^F-PSMA-1007 (24) could induce a risk of false-positive skeletal findings.

## CONCLUSION

We have presented the first—to our knowledge—large prospective trial comparing ^18^F-PSMA PET/CT and ^18^F-NaF PET/CT in patients with newly diagnosed high-risk PC. Both imaging modalities demonstrated high diagnostic accuracy for detecting bone metastases, with no significant difference between them. ^18^F-NaF PET/CT offers no additional value in patients for whom ^18^F-PSMA PET/CT does not identify bone metastases.

## DISCLOSURE

No potential conflict of interest relevant to this article was reported.

## Notes

KEY POINTS **QUESTION:** Is ^18^F-PSMA PET/CT sufficient for detecting bone metastases in patients with newly discovered PC? **PERTINENT FINDINGS:** This prospective study comprised 160 patients and demonstrated high diagnostic accuracies for both ^18^F-PSMA PET/CT and ^18^F-NaF PET/CT, with no significant difference between the two modalities. **IMPLICATIONS FOR PATIENT CARE:** If no bone metastases are detected on ^18^F-PSMA PET/CT, additional ^18^F-NaF PET/CT may be redundant.
